# Methylation of BDNF gene in association with episodic memory in women

**DOI:** 10.3389/fnins.2023.1092406

**Published:** 2023-03-16

**Authors:** Ivana Alece Arantes Moreno, Daniela Rodrigues de Oliveira, Aline Ribeiro Borçoi, Luciana Fungaro Rissatti, Flávia Vitorino Freitas, Lidia Maria Rebolho Batista Arantes, Suzanny Oliveira Mendes, Tamires dos Santos Vieira, Bárbara Risse Quaioto, Paola Cerbino Doblas, Amanda Sgrancio Olinda, Ester Ribeiro Cunha, Joaquim Gasparini dos Santos, Júlia Assis Pinheiro, Bruna Pereira Sorroche, Adriana Madeira Alvares da Silva

**Affiliations:** ^1^Biotechnology/Renorbio Postgraduate Program, Universidade Federal do Espírito Santo, Vitória, ES, Brazil; ^2^Department of Pharmacology and Toxicology, Medical College of Wisconsin, Milwaukee, WI, United States; ^3^Departamento de Patologia, Universidade Federal de São Paulo, São Paulo, SP, Brazil; ^4^Natural Products and Derivatives Laboratory (LIM-26), Department of Surgery, University of São Paulo Medical School, São Paulo, SP, Brazil; ^5^Departamento de Farmácia e Nutrição, Universidade Federal do Espírito Santo, Alegre, ES, Brazil; ^6^Molecular Oncology Research Center, Barretos Cancer Hospital, Barretos, São Paulo, Brazil; ^7^Departamento de Morfologia, Universidade Federal do Espírito Santo, Vitória, ES, Brazil

**Keywords:** BDNF, DNA methylation, episodic memory, women, Rey Auditory Verbal Learning Test (RAVLT)

## Abstract

Brain-derived neurotrophic factor (BDNF) gene regulation plays an important role in long-term memory formation, and the DNA methylation (DNAm) level of *BDNF* promoters has been associated with episodic memory deficits. Our aim was to explore the association between DNAm levels in *BDNF* promoter IV with verbal learning and memory performance in healthy women. We conducted a cross-sectional study by recruiting 53 individuals. Episodic memory was assessed by using the Rey Auditory Verbal Learning Test (RAVLT). Clinical interviews, RAVLT, and blood sample collection were assessed in all participants. DNAm was measured on DNA from whole peripheral blood using pyrosequencing. According to generalized linear model (GzLM) analyses, cytosine guanine dinucleotide (CpG) site 5 showed significant associations between learning capacity (LC, *p* < 0.035), that is, every 1% of DNA methylation at CpG site 5 results in a 0.068 reduction in verbal learning performance. To the best of our knowledge, the current study is the first to show that *BDNF* DNAm plays an important role in episodic memory.

## 1. Introduction

Throughout life, the brain-derived neurotrophic factor (BDNF) plays an important role in neural development and synaptic plasticity in learning and memory (reviewed by Day and Sweatt, 2010; Silakarma and Sudewi, 2019; Azman and Zakaria, 2022). The BDNF gene is a member of the neurotrophin family that is highly expressed in the central nervous system (CNS) and has impacts on many brain and body functions. The human BDNF gene is located on chromosome 11 and in the p13–14 region and contains ~ 70 kb, including 11 exons and nine different promoters that regulate its expression (Pruunsild et al., 2007). In addition, they have alternative splicing sites and the possibility of formation in brain-specific transcripts and other non-brain tissues (Pruunsild et al., 2007; Notaras and van den Buuse, 2019).

The BDNF gene can be regulated through epigenetic modifications, including DNA methylation. Studies suggest that BDNF methylation may influence gene regulation by repressing expression and reducing BDNF protein levels. Different promoters regulate BDNF in complex ways, and its expression is also modulated by endogenous and exogenous stimuli, such as stress and physical activity (Pruunsild et al., 2007; Watts et al., 2018; Notaras and van den Buuse, 2020).

Other factors influencing BDNF methylation include tobacco consumption, antidepressant drugs, and early life adversity stress (Lieb et al., 2018; Fachim et al., 2021; Abdelkhalek et al., 2022). Previous studies report sex differences in protein levels and gene methylation in BDNF, potentially mediated by estrogen signaling (Lommatzsch et al., 2005; Chan and Ye, 2017).

BDNF is essential in synaptic plasticity and is related to human memory and learning function, especially for neuroplasticity in the hippocampus and prefrontal cortex (Leal et al., 2014; Cocco et al., 2018). Recent studies have suggested that the dysregulation of BDNF contributes to the pathogenesis of several major diseases and disorders, such as Huntington's disease, Alzheimer's disease, and depression (reviewed by Zheleznyakova et al., 2016), especially mediated by epigenetic processes. Some studies show that BDNF methylation CpG regions have been linked to cognitive deficits and psychiatric diseases (Grayson and Guidotti, 2013; Azman and Zakaria, 2022).

Moreover, differences are observed in the methylation level at CpG sites of BNDF promoter IV on poor performance in learning and memory tasks such as the Brief Visuospatial Memory Test-Revised (BVMT-R) (Engelmann et al., 2020) and the Rey Complex Figure Test (RCFT) in women with major depressive disorders (MDDs) (Ferrer et al., 2019). There is increasing evidence for the involvement of epigenetic regulation of the BDNF in cognition. However, to our knowledge, no previous studies have addressed the involvement of BDNF methylation on episodic memory in women without MDD.

The aim was to explore the association of cytosine guanine dinucleotide (CpG) sites within the BDNF gene promoter on episodic memory in women. With this background, we hypothesized that the specific methylation of promoter regions of the BDNF IV would be associated with components of episodic memory related to immediate memory (IM), learning capacity (LC), immediate recall (IR), delay recall (DR), and delay recognition (DRec).

## 2. Methods

### 2.1. Study design

A cross-sectional study was conducted among adult Brazilian women recruited from the primary care of the Brazilian Unified Health System (in Portuguese, Sistema Único de Saúde—SUS). The neuropsychological testing data, demographic, clinical, and lifestyle information, and blood samples were collected at similar time points.

### 2.2. Procedure

Participants were recruited from Brazilian Unified Health System (SUS) in Alegre City, located in Espírito Santo State, southeastern Brazil, between April and July 2017. All participants were selected from a PPSUS project (Research Program for SUS) under number 74713515/2016. Study procedures were approved by the Research Ethics Committee of the Universidade Federal do Espírito Santo (CEP/CCS/UFES), Brazil (Brazil Number: 3.420.734 - CAAE:08454919.5.0000.8151) in June, 2019. All participants in the study were required to sign written informed consent forms (ICFs). Inclusion criteria were as follows: (1) women aged between 20 and 59 years and (2) living in urban areas, being the only study participant in the residence, and being literate. Exclusion criteria were as follows: (1) the presence of medical conditions or medication use that might affect inflammatory pathways; (2) a psychiatric condition that poses life-risk and clinical comorbidities that are potentially life-threatening, such as psychosis or suicidal ideation at the moment of study. Those subjects who met the inclusion/exclusion criteria and gave their written informed consent before baseline procedures were subsequently included in this study.

### 2.3. Participants

A total of 103 women were eligible for screening; of whom, 53 were excluded. The reasons for exclusion were (a) not enough DNA (*n* = 4) and (b) no signal on pyrosequencing data (*n* = 6). A total of 43 women declined to participate in this study for personal reasons (family emergence and/or time).

### 2.4. Neuropsychological assessment

The Brazilian version of the Rey Auditory Verbal Learning Test (RAVLT) was used to assess different components of episodic memory (de Paula and Malloy-Diniz, 2019). RAVLT is sensitive to verbal learning and memory deficits and was conducted and reviewed by a certified neuropsychologist with more than 5 years of experience and was performed in a quiet room. A 15-word noun list (List A) was read aloud to the participants by a researcher with experience in the application of neuropsychological assessment to validate participants according to the Brazilian manual's instructions, in a fixed order and with a 1-s interval between words, for five consecutive trials. Each of the five trials was followed by a free recall test, for which the participant was asked to repeat as many words as possible in any order. After the completion of Trial 5, a second (new) list of 15 words (List B; interference list) was read aloud. Immediately after the free recall test of List B, and without an additional presentation of List A, an immediate recall of List A (Trial 7) was assessed. After a 20-min delay period (with no other verbal memory tests administered during this interval), the delay recall of List A (Trial 8) was assessed. Immediately after Trial 8, a delayed recognition test was administered by providing the participant with a matrix of 50 words containing both List A and List B words, in addition to 20 words that were phonemically or semantically similar to those in both lists and requesting only the identification of List A words.

### 2.5. Study measures

Data collection was performed through an individual interview with a semi-structured questionnaire that evaluated sociodemographic and clinical characteristics, which were elaborated based on the Individual and Domiciliary Registry Files, Ministry of Health, Brazil. Individuals were categorized according to age, ethnicity, relationship status, schooling, per capita income classification, alcohol, leisure activity, and smoking. In addition, anthropometric data were collected to calculate body mass index (BMI).

### 2.6. Depression assessment

The evaluation of depressive symptoms was obtained from the Beck Depression Inventory-II (BDI-II), a self-reported instrument to evaluate the presence and severity of depressive symptoms with Brazilian validation (Beck et al., 1996; Gomes-Oliveira et al., 2012). The questionnaire contains 21 affirmation groups related to the participant's feelings in the past 2 weeks, counting with the day of application, varying in intensity from 0, 1, 2, or 3. The total score is the sum of the score of each question.

### 2.7. Biological measurements

#### 2.7.1. DNA extraction and methylation analysis

Venous blood was obtained from all participants and collected in EDTA tubes. DNA was extracted according to Salazar et al. (1998). NanoDrop assessed DNA concentrations, quantity, and quality.

We selected the region IV exon of the brain-derived neurotrophic factor (BDNF) gene covering nine target sites for methylation analysis (chr11: 27,701,519 to 27,701,826 GenBank NCBI—Access number: NC_000011.10). The exon IV promoter region of the BDNF gene was examined in this study. The region included the sequence where the transcription factor cyclic AMP-responsive element-binding protein (CREB-binding) and the CpG sites analyzed correspond to CpGs 5 to 11, containing 13 CpG sites. The primer sequences of the CpG sites were relative to the region analyzed previously by Braithwaite et al. (2015) and Kundakovic et al. (2015).

According to the manufacturer's recommendations, genomic DNA was converted to bisulfite using the EZ^®^ DNA Methylation Gold Kit (Zymo Research, Irvine, CA.). The pyrosequencing of bisulfite-treated genomic DNA methylation analysis was performed by the PSQ96ID Pyrosequencer (Qiagen^®^, Valencia, CA) with reagents PyroMark Gold Q96 (Qiagen^®^, Valencia, CA) according to manufacturer's protocol. A total of two sequencing primers were used due to the size of the amplicon. The primer sequence analyzed was relative to the region analyzed previously by Braithwaite et al. (2015). The analyzed region, PCR primers, conditions, and pyrosequencing primers are described in Supplementary Table 1.

### 2.8. Statistical analyses

The Shapiro–Wilk normality test was used to examine normality. The socioeconomic and lifestyle variables were presented at relative and absolute frequencies and percentages (%). The continuous variables were presented in mean and standard deviation (SD) or median and interquartile interval (IR).

Generalized linear models (GzLMs) were used to determine the influence of CpG sites of BDNF gene promoter in the components of episodic memory. The dependent variables included in the models were scores of immediate memory, learning capacity, immediate recall, delay recall, and delay recognition. Dependent variables were not normally distributed; in GzLM models, the distribution was *gamma*. In all the models, only the main effects were determined.

Statistical analyses were performed using SPSS version 26.0 (IBM Corp., Armonk, NY, USA). Inter-individual variability (age, education, BDI-II, BMI, current antidepressant use and lifestyle, current smoking, current alcohol consumption, and physical activity) was chosen to adjust all the GzLM models. Akaike's information criterion (AIC) was used to select the best model in the analysis. GzLM analysis was conducted separately for each outcome to optimize the chances of convergence. The confidence level of 95% was applied, and a significance of 5% (*p* < 0.05) was considered. *p*-value was adjusted by Bonferroni method.

## 3. Results

### 3.1. Sociodemographic, lifestyle, physical characteristics, cognition performance, and DNA methylation profile

The interindividual, lifestyle, and physical characteristics of this sample are presented in Table 1. Women (with age between 20 and 59 years) showed that the prevalence of overweight and obesity was 66.0% (BMI mean = 28.4 Kg/m^2^ and SD = 5.95), and the prevalence of symptoms of depression was 17%. The income/month was R$937.00 (corresponding to US$1.00, the value of R$3.23 in the study period). Income was classified according to the Center Social Policies for Getúlio Vargas Foundation (FGV) (Neri, 2008), which considered low-income individuals as having a per capita income of <$5.00/per day.

**Table 1:** Characterization of all participants.

**Characteristics**	**All (*n* = 53)**
**Age (years old)**	
Age, median (IR)	47.5 (19)
**Education**, ***n*** **(%)**	
<9 years of study	21 (39.6)
≥9 years of study	32 (60.4)
**Clinical, physical and lifestyle characteristics**	
**Psychological health**, ***n*** **(%)**	
BDI score, median (IR)	7.5 (11)
BDI <18, *n* (%)	41 (77.3)
BDI ≥ 18, *n* (%)	9 (17.0)
BDI not available^#^	3 (5.7)
**Antidepressant medication**, ***n*** **(%)**	
No	50 (94.3)
Yes	3 (5.7)
BMI, mean (±SD)	28.4 (5.95)
Underweight/Normal, *n* (%)	18 (34.0)
Overweight, *n* (%)	15 (28.3)
Obese, *n* (%)	20 (37.7)
**Alcohol**, ***n*** **(%)**	
Non-alcohol	37 (69.8)
Alcohol	16 (30.2)
**Smoking**, ***n*** **(%)**	
Non-smoker	51 (96.2)
Smoker	2 (3.8)
**Physical activity**, ***n*** **(%)**	
Non-physical activity	32 (60.4)
Physical activity	21 (39.6)

Quantitative variables presented in medians and interquartile ranges (IR) or means and standard deviations (±SD) according to normality (Shapiro-Wilk test); BDI-II, Beck Depression Inventory II; BMI, Body Mass Index; ^#^Not available (not considered in the statistical calculations).

Regarding cognition analyses based on RAVLT performance, women showed lower cognitive performance in the 41–51 age group than the other groups in the variables such as immediate memory, learning capacity, immediate recall, and delayed recall.

### 3.2. BDNF CpG 5 methylation shows a reduction in verbal memory performance GzLM

To assess the relationship between the aspects of episodic memory and all methylation of CpGs of BDNF IV promoter regions, we also performed GzLM models, with each RAVLT variable as the dependent, and all CpGs and the confounding factors such as age, BMI, BDI-II score, education, current antidepressive use, current smoking, current alcohol consumption, and physical activity were considered as independent variables.

The results showed that the CpG site 5 remained as a risk factor for learning capacity (B = −0.068; Wald CI = −0.118 to −0.019; *p*-value adj. = 0.035), indicating that every 1% of DNA methylation at the CpG site 5 results in a 0.068 reduction in verbal learning performance. No differences were found in CpG sites 6 to 11 with other components of episodic memory (*p* > 0.05, Table 2).

**Table 2:** GzLM results for models with all CpGs, ajusted by confounding factors: age, education, current andidepressive use, BMI, BDI-II scores, current smoking, current alcohol consumption, and physical activity.

**RAVLT variables**	**CpG sites**	**Parameter estimates**					
		**Estimate**	**Std. Error**	**95% CI**	**Wald Chi-square**	***p*** **-value**	***p*** **-value adj** ^*^
IM	5	−0.071	0.0355	−0.141 – −0.002	4.032	0.045	0.225
	6	0.057	0.0429	−0.027 – 0.141	1.752	0.186	0.930
	7	0.015	0.0295	−0.043 – 0.073	0.26	0.61	>0.999
	8	−0.01	0.0322	−0.074 – 0.053	0.103	0.748	>0.999
	9	−0.011	0.0163	−0.043 – 0.021	0.457	0.499	>0.999
	10	0.015	0.0427	−0.069 – 0.099	0.124	0.725	>0.999
	11	−0.005	0.0111	−0.027 – 0.017	0.216	0.642	>0.999
LC	5	−0.068	0.0252	−0.118 – −0.019	7.381	0.007	0.035
	6	−0.014	0.0305	−0.074 – 0.046	0.208	0.649	>0.999
	7	0.007	0.0217	−0.035 – 0.05	0.105	0.745	>0.999
	8	0.003	0.0232	−0.043 – 0.048	0.014	0.905	>0.999
	9	−0.007	0.0117	−0.03 – 0.016	0.352	0.553	>0.999
	10	0.059	0.0301	0 – 0.118	3.799	0.051	0.255
	11	0.006	0.0077	−0.009 – 0.021	0.582	0.446	>0.999
IR	5	−0.081	0.0365	−0.153 – −0.01	4.959	0.026	0.130
	6	−0.004	0.0432	−0.088 – 0.081	0.007	0.934	>0.999
	7	0.006	0.0325	−0.057 – 0.07	0.038	0.845	>0.999
	8	−0.059	0.0338	−0.126 – 0.007	3.08	0.079	0.395
	9	0.028	0.019	−0.009 – 0.065	2.14	0.144	0.720
	10	0.063	0.044	−0.023 – 0.15	2.069	0.15	0.750
	11	0.006	0.0106	−0.015 – 0.026	0.273	0.602	>0.999
DR	5	−0.09	0.0455	−0.179 – −0.001	3.9	0.048	0.240
	6	−0.012	0.0537	−0.117 – 0.093	0.052	0.82	>0.999
	7	0.016	0.0399	−0.062 – 0.095	0.171	0.679	>0.999
	8	−0.057	0.0431	−0.142 – 0.027	1.775	0.183	0.915
	9	0.023	0.0239	−0.024 – 0.069	0.89	0.346	>0.999
	10	0.038	0.0533	−0.066 – 0.143	0.509	0.476	>0.999
	11	0.008	0.0134	−0.018 – 0.034	0.35	0.554	>0.999
DRec	5	−0.046	0.0203	−0.086 – −0.006	5.089	0.024	0.120
	6	−0.027	0.0246	−0.075 – 0.022	1.165	0.28	>0.999
	7	0.002	0.0165	−0.03 – 0.035	0.018	0.894	>0.999
	8	0.017	0.0176	−0.018 – 0.051	0.897	0.344	>0.999
	9	−0.003	0.0093	−0.022 – 0.015	0.12	0.729	>0.999
	10	0.011	0.0247	−0.038 – 0.059	0.188	0.664	>0.999
	11	0.004	0.0061	−0.008 – 0.016	0.445	0.505	>0.999

IM, Immediate Memory; LC, Learning Capacity; IR, Immediate Recall; DR, Delayed Recall; DRec, Delay Recognition. ^*^p-value adjusted by Bonferroni method.

## 4. Discussion

We have studied the association of BDNF DNA methylation with episodic memory in healthy Brazilian women. This study presents the relevance of BDNF gene regulation in association with the acquisition (learning capacity) of episodic memory. Our main finding shows that DNA methylation at CpG site 5 on BDNF gene promoter IV is associated with poor verbal learning performance. In addition, every 1% of DNA methylation at CpG site 5 increases the risk factor for poor performance in learning capacity in the RAVLT test in women.

There are no reports in the literature on BDNF DNA methylation and episodic memory in healthy women. However, investigations on epigenetic changes in BDNF have been described in individuals with psychiatric disorders and neurodegenerative diseases, including depression, Huntington's disease, and schizophrenia (Ikegame et al., 2013; Zheleznyakova et al., 2016; Di Carlo et al., 2019; Gutierrez et al., 2020). Engelmann et al. (2020) focused on the promoter region of exon IV of the BDNF gene, however, they have investigated a different sequence than the one analyzed in this study. They found no association between methylation status in the BDNF exon IV promoter and memory test performance in depressive patients on antidepressant therapy. Conversely, Ferrer et al. (2019) found an association between methylation of the exon IV promoter region of the BDNF gene and memory.

Interestingly, in the study by Ferrer et al. (2019), patients with major depressive disorder (MDD) showed an association between BDNF promoter IV methylation at CpG sites 5, 9, and 10 and poorer cognition in several cognitive domains (verbal and visual learning and memory, working memory, processing speed, and executive functioning). Our results support the evidence that a higher methylation level of CpG site 5 is associated with poorer cognitive performance in different neuropsychological verbal learning and memory (episodic memory) assessments.

Interestingly, BDNF promoter IV contains a binding site for the transcription factor cyclic AMP-responsive element-binding protein (CREB) and is known to be sensitive to DNA methylation changes in animals (Braithwaite et al., 2015; Kundakovic et al., 2015) and clinical models (Ikegame et al., 2013; Zheleznyakova et al., 2016; Di Carlo et al., 2019; Ferrer et al., 2019; Gutierrez et al., 2020). CREB plays a central role in the molecular mechanism underlying learning and memory and regulates BDNF transcriptional activity. Epigenetic changes (i.e., increase in methylation level) within the CREB-binding site affect gene regulation by decreasing BDNF gene and protein expression, promoting learning and memory deficits (Ikegame et al., 2013; Zheleznyakova et al., 2016; Sabatucci et al., 2020). This finding is perhaps not surprising because the BNDF IV in the adult brain is regulated to DNAm in the response of neural plasticity, and it affects the acquisition of memory (learning capacity) in humans. Further studies are needed to understand the relationship between BDNF and episodic memory.

Other authors have evaluated BDNF protein levels and their relationship with memory, with contradictory results. Wilkosc et al. (2016) found no strong and significant relationship between serum BDNF concentrations (proBDNF and mBDNF) and episodic memory performance by RAVLT in healthy adult individuals. The authors suggested that the impact of the effect of mBDNF is greater in elderly individuals with cognitive decline and neurodegenerative and psychiatric diseases. In addition, they hypothesized that BDNF has a greater influence on spatial memory than episodic memory. Contrasting, the study by Siuda et al. (2017), when assessing the involvement of serum BDNF levels and neurodegenerative diseases, found significant results and proposes that serum BDNF levels possibly are dependent on a neurodegenerative process in Alzheimer's disease. In the same study, the authors report a positive correlation between serum BDNF levels and episodic memory but found no differences for a single cognitive domain. Thus, further studies are needed to understand the relationship between BDNF and episodic memory.

There are some limitations to our study that merit discussion. First, the small sample size of our study might have reduced the statistical power to detect a small effect size. Second, the cross-sectional design of the study does not allow causal inferences. Third, we did not address sex differences. Fourth, education was retrospectively self-reported and was not counted by the number of years of the study. Fifth, we did not assess BDNF expression and protein. This is the first study and provides an important basis for more extensive research. Therefore, future studies with larger samples and with men are needed to evaluate the hypothesis that individuals with higher methylation levels have greater difficulty in the cognitive processes of memory. This study provides a basis for understanding the role of regulation by BDNF exon IV DNA methylation in episodic memory. Therefore, we can evidence an involvement between BDNF gene methylation and episodic memory performance. Future studies evaluating the impact of epigenetic changes on episodic memory, including their influence on BDNF gene and protein expression, are needed to understand these complex relationships better.

## Data availability statement

The raw data supporting the conclusions of this article will be made available by the authors, without undue reservation.

## Ethics statement

The studies involving human participants were reviewed and approved by Research Ethics Committee of the Universidade Federal do Espírito Santo (CEP/CCS/UFES), Brazil (Number: 3.420.734 - CAAE:08454919.5.0000.8151). The patients/participants provided their written informed consent to participate in this study.

## Author contributions

DR and AM contributed to the study design. IA, AR, FV, AS, JA, JG, and ER contributed to the acquisition of data. IA, AR, LF, SO, FV, LA, DR, JG, and AM assisted with data analysis and interpretation of findings. IA, AR, JG, JA, SO, TS, BR, PC, BP, LA, DR, and AM contributed with reagents or materials or analysis tools. IA, AR, SO, DR, and AM drafted the manuscript. All authors critically reviewed the manuscript and read and approved the final manuscript.
