# Supplementary material for: Methylation of BDNF gene in association with episodic memory in women
Source: Front Neurosci. 2023 Mar 16;17:1092406. doi: 10.3389/fnins.2023.1092406 (PMC10060857; doi:10.3389/fnins.2023.1092406)

Supplementary material: Q-Q plots and residuals.

Immediate Memory (RAVLT) outcome

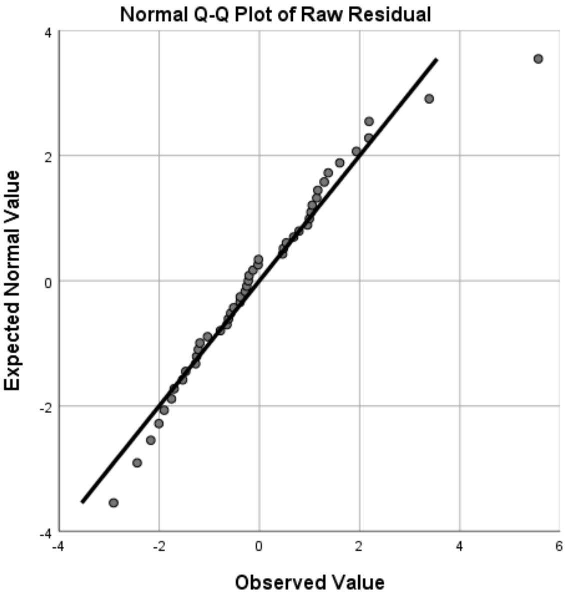

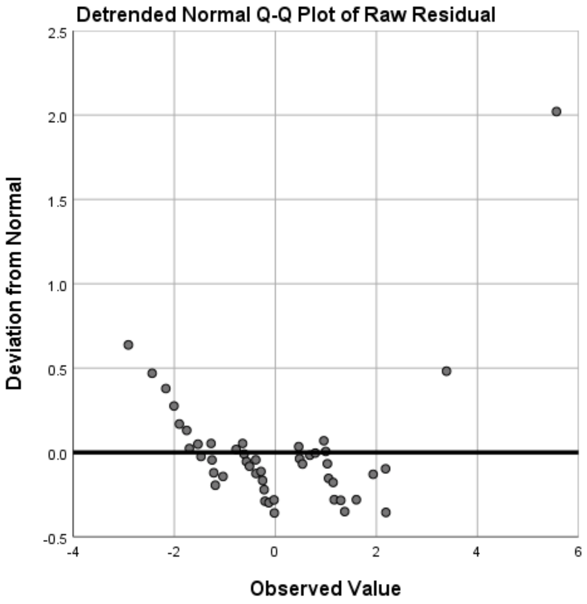

Learning Capacity (RAVLT) outcome

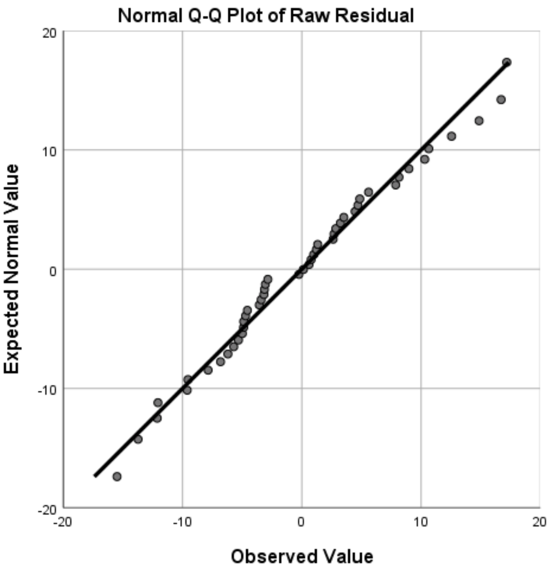

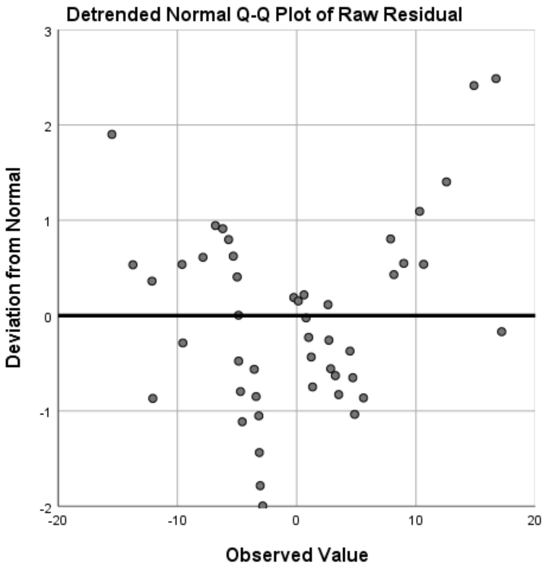

Immediate Recall(RAVLT) outcome

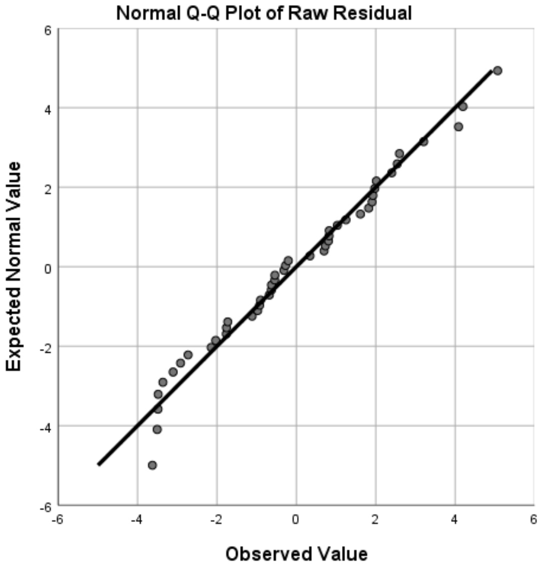

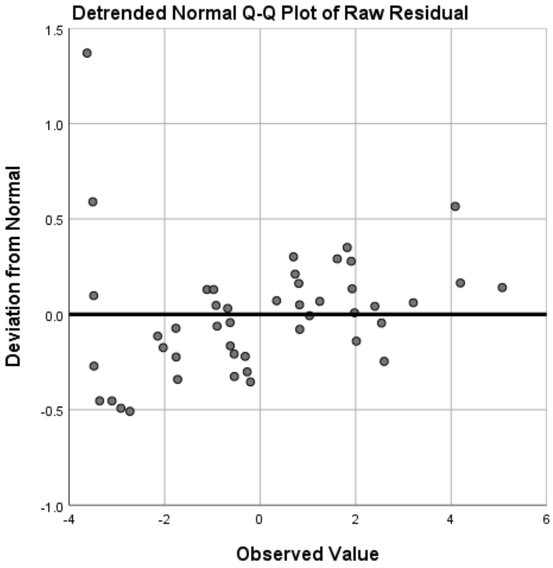

Delay Recall (RAVLT) outcome

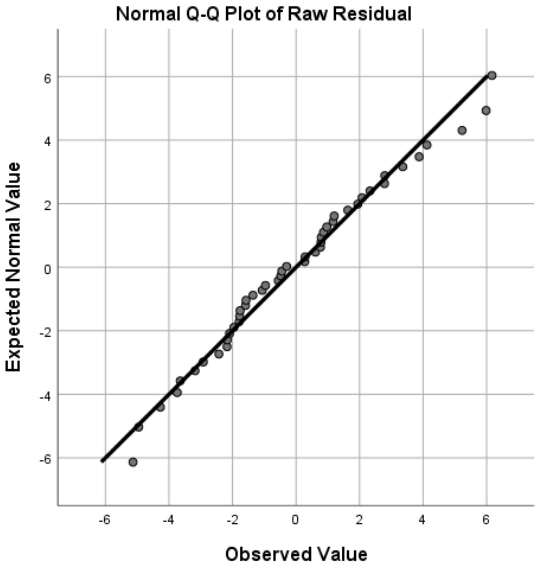

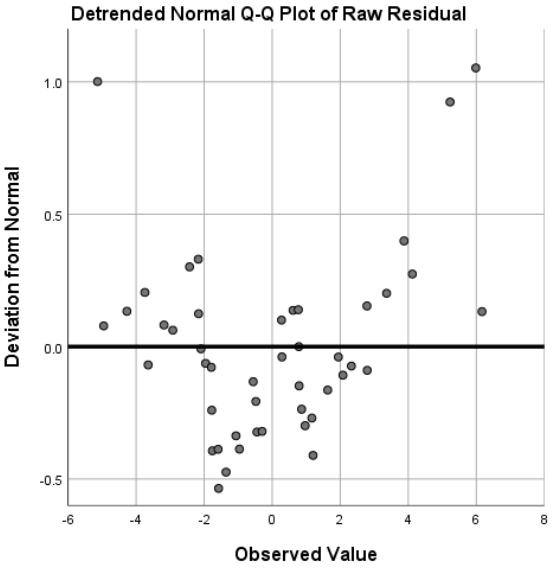

Delay Recognition (RAVLT) outcome

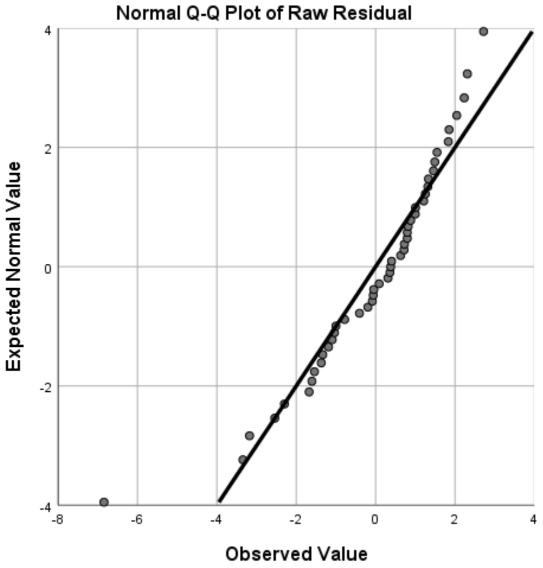

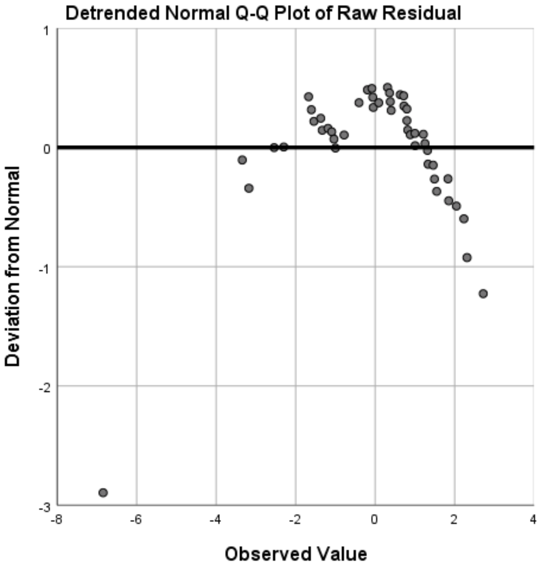

Supplement: Supplementary file 2 [file Image_1.pdf]
